# Use of Medicinal Cannabis and Synthetic Cannabinoids in Post-Traumatic Stress Disorder (PTSD): A Systematic Review

**DOI:** 10.3390/medicina55090525

**Published:** 2019-08-23

**Authors:** Laura Orsolini, Stefania Chiappini, Umberto Volpe, Domenico De Berardis, Roberto Latini, Gabriele Duccio Papanti, John Martin Corkery

**Affiliations:** 1 Psychopharmacology, Drug Misuse and Novel Psychoactive Substances Research Unit, School of Life and Medical Sciences, University of Hertfordshire, Hatfield, AL10 9AB Herts, UK; 2 Neomesia Mental Health, Villa Jolanda Hospital, 60035 Jesi, Italy; 3 Polyedra, 64100 Teramo, Italy; 4 Department of Clinical Neurosciences/DIMSC, School of Medicine, Section of Psychiatry, Polytechnic University of Marche, 60121 Ancona, Italy; 5 NHS, Department of Mental Health, Psychiatric Service of Diagnosis and Treatment, Hospital “G. Mazzini”, 64100 ASL 4 Teramo, Italy; 6 Department of Neuroscience, Imaging and Clinical Science, Chair of Psychiatry, University of “G. D’Annunzio”, 66100 Chieti, Italy

**Keywords:** PTSD, trauma, CBD, cannabis, endocannabinoid system, cannabinoids, synthetic cannabinoids

## Abstract

*Background and Objectives*: Post-traumatic stress disorder (PTSD) is a common psychiatric disorder resulting from a traumatic event, is manifested through hyperarousal, anxiety, depressive symptoms, and sleep disturbances. Despite several therapeutic approaches being available, both pharmacological and psychological, recently a growing interest has developed in using cannabis and synthetic cannabinoids stems from their consideration as more efficient and better tolerated alternatives for the treatment of this condition. The present paper aims to evaluate the clinical and therapeutic potentials of medical cannabis and synthetic cannabinoids in treating PTSD patients. *Methods*: A systematic electronic search was performed, including all papers published up to May 2019, using the following keywords (((cannabis[Title/Abstract]) OR (synthetic cannabinoids [Title/Abstract])) AND ((PTSD[Title/Abstract]) OR (Posttraumatic stress disorder[Title/Abstract]))) for the topics ‘Cannabis’, ‘Synthetic Cannabinoids’, ‘PTSD’, and MESH terms, on the PubMed, Cochrane Library, and Web of Science online databases. For data gathering purposes, PRISMA guidelines were followed. Results were organized into two groups, considering cannabis and synthetic cannabinoids as different therapeutic approaches for PTSD. *Results*: Present data show that cannabis and synthetic cannabinoids, both acting on the endocannabinoids system, may have a potential therapeutic use for improving PTSD symptoms, e.g., reducing anxiety, modulating memory-related processes, and improving sleep. *Conclusions*: Even though the current literature suggests that cannabis and synthetic cannabinoids may have a role in the treatment of PTSD, there is currently limited evidence regarding their safety and efficacy. Therefore, additional research is needed in order to better understand the effectiveness and therapeutic usage of these drug classes and monitor their safety.

## 1. Introduction

### 1.1. Post Traumatic Stress Disorder (PTSD)

Post-traumatic stress disorder (PTSD) is a psychiatric condition that develops as an aberrant adaptation to a traumatic event. The disorder may manifest itself through a broad range of symptoms, involving cognition (e.g., repeated recall of the event, through intrusive thoughts, flashbacks, nightmares), mood (e.g., depression, anxiety), and emotion (e.g., psychological instability, impulsivity, and hyperarousal), and impaired social abilities [1,2], that can cause a significant alteration in personal and interpersonal functioning. Thus, the conceptual model of PTSD as a disorder of fear conditioning and extinction can assist our understanding of how the underlying neurobiological dysfunction observed in subjects suffering from PTSD. Symptoms arise in response to an acutely traumatic event, that is an incident that may cause serious injury or is life-threatening and is perceived as uncontrollable and dangerous. The effect is an activation of the hypothalamic–pituitary axis as well as of the locus coeruleus and the noradrenergic system, regions which are connected to the amygdala and hippocampus, mediating, respectively, fear conditioning and memory consolidation [3]. As a result, the experience, associated sensory stimuli, and emotional response (fear) become encoded, such that later exposure to a related cue triggers reactivation of the traumatic memories as well as anxiety and increased arousal [3]. Thus, the pathophysiology of PTSD involves several neurotransmitters, including the noradrenergic, serotonergic, endogenous cannabinoid, and opioid systems, as well as the hypothalamic–pituitary adrenal axis and the release of the corticotropin-releasing factor, which are systems involved in other psychiatric conditions, such as mood or anxiety disorders [4]. Structural changes and central neurotransmitter imbalances involve the following mechanisms: (1) increased responsivity in the amygdala and decreased volume in the prefrontal cortex and in the hippocampus, triggering hyperarousal and anxiety; (2) dysfunction in the hypothalamic–pituitary axis coordinating the neuroendocrine stress response systems; and (3) dysregulation of neurotransmitters, e.g., increase in norepinephrine and glutamate, and decrease in serotonin [3,4,5].

### 1.2. Therapeutic Approaches for PTSD

Pharmacotherapy approved for PTSD includes traditional antidepressant and anxiolytic medications, e.g., selective serotonin reuptake inhibitors (SSRIs), such as sertraline, paroxetine, fluoxetine; and serotonin and norepinephrine reuptake inhibitors (SNRIs), such as venlafaxine, which are considered as first-line treatments [5,6]. However, both SSRIs and SNRIs have only partial efficacy, with remission rates reported to range from 20% to 30%, as well as the onset of potential side-effects, mainly responsible for their early discontinuation and consequent poor efficacy [7]. Furthermore, second-generation antipsychotics (SGAs)—such as risperidone, quetiapine, and olanzapine—are suggested for managing symptoms for adults with a diagnosis of PTSD in a secondary care setting [6]. According to current guidelines, a trauma-focused psychotherapy (TFP), acting on cognitive restructuring is primarily recommended [8]. Finally, cognitive behavioral therapy (CBT) and eye movement desensitization and reprocessing (EMDR) have been shown to be effective in the treatment of PTSD and trauma-related disorders, by acting on the dysregulation of the learning process of aversive memories [6].

Other promising approaches for PTSD treatment are represented by molecules acting on different neurotransmitter circuits, e.g., (1) prazosin, which is an alpha 1 adrenergic antagonist, found to be effective in reducing symptoms of anxiety, hyperarousal and sleep disorders, which are typical of PTSD; (2) gamma amino butyric acid (GABA) agonists, such as pregabalin or gabapentin, which may be used in addition to an antidepressant therapy; (3) N-methyl d-aspartate (NMDA) receptor partial agonists such as D-cycloserine (DCS), which has been associated with a reduction in symptoms of anxiety, avoidance, and numbing, and has also demonstrated an ability to augment the learning process for extinction of conditioned fear responses in both animal and human models [3,9].

### 1.3. Cannabis use in PTSD: A Coping Strategy?

Research has demonstrated a strong link between trauma, PTSD, and substance use disorders (SUDs) in general, and particularly between cannabis use disorders and PTSD [10,11,12,13]. In fact, the pathophysiology of PTSD involves many of the structures and neurocircuitry identified as key components in the development and/or perpetuation of addictive processes, such as amygdala hyperactivity, chronic activation of brain stress systems, and increased corticotropin-releasing factor (CRF) during acute drug-withdrawal; and the medial prefrontal cortex and its connections to the nucleus accumbens and ventral pallidum involved in animal models of craving and drug-induced reinstatement [3]. It has been estimated that individuals with PTSD are 2–4 times more likely to have a SUD compared to individuals without PTSD [14]. An association between PTSD and SUD has been documented amongst US veterans [13,15,16], possibly due to shared etiological factors, but also in an attempt to act as a copying strategy for PTSD symptoms using substances, and specifically cannabis, as a self-medication [3,10,11,13,17]. In fact, cannabis has been consumed for to its calming and relaxing effects, in order to cope with symptoms of intrusions (like repeated and disturbing thoughts or dreams) and hyperarousal (anxiety, trouble sleeping, irritability) [1,7,18,19,20,21,22]. Between 2002 and 2009, the diagnosis of a cannabis use disorder increased more than 50% (from 0.66% to 1.05%) amongst veterans [15], facilitated by users’ perception of safeness compared to psychopharmacological compounds and/or alcohol, both of which are reported to have undesired side-effects, ranging from loss of cognitive acuity and social withdrawal to anhedonia and decreased sex drive/libido, which cannabis usually did not cause [23].

### 1.4. Cannabis, Cannabinoids, and Their Role in PTSD

Marijuana is derived from the Sativa and Indica species of the Cannabis plants. It contains cannabinoids and several other classes of chemical compounds acting on the cannabinoid receptors. Specifically, Δ-9-tetrahydrocannabinol (THC) is a highly lipophilic alkaloid and is the primary psychoactive ingredient in marijuana, varying from 0.2% to 30% potency per plant and strain; whilst cannabidiol (CBD) is a non-psychotomimetic cannabinoid, with neuroprotective, analgesic, sedative antiemetic, antispasmodic, anti-inflammatory, and anxiolytic properties [7]. Both THC and CBD act on cannabinoid receptors, but, compared with THC, CBD shows a lower CB_1_ and CB_2_ receptor affinity and, being an inverse agonist at the human CB_2_ receptor, it shows anti-inflammatory effects as well [24]. The cannabinoid receptors are part of the endocannabinoid system (eCS) together with endogenous cannabinoids, such as N-arachidonoyl ethanolamine (anandamide) and 2-arachidonoyl glycerol (2-AG) [4,7]. CB_1_ receptors are mainly located in the brain and widely expressed in the prefrontal-limbic system, including areas such as the amygdala, hippocampus, and prefrontal cortex, while CB_2_ receptors are mainly expressed in peripheral immunological tissue, although their presence in the central nervous system has been also recently documented in regions such as the amygdala, hippocampus, striatum, substantia nigra and cortex. Both cannabinoid receptors cause different molecular events, resulting in a general inhibition of neurotransmitters release, such as glutamate, serotonin, noradrenaline, and dopamine, from pre-synaptic terminals of neurons where cannabinoid receptors are expressed [7,25]. In fact, the activation of circuitries and mechanisms involving CB receptors may intervene in some PTSD neurobiological pathways and symptoms, influencing its etiology and maintenance, e.g.:

(1) CB_1_ receptors are found in moderate to high levels throughout brain limbic structures, and have been shown to possess modulating properties on behaviors, including mood, stress, learning, and memory [4]: in fact, by activating the CB_1_ receptors in the amygdala, cannabis can potentially block the consolidation of aversive memories, fear, and anxiety; moreover, through stimulating CB_1_ receptors in the prefrontal cortex, cannabis may increase serotonin and, therefore, display antidepressant properties; finally, cannabis agonism on CB_1_ receptors in the hippocampus seems to improve neurogenesis, mood, and memory as well as causing decreases in hypervigilance, hyperarousal, and intrusive memories, effects which may contribute to the anxiolytic and antidepressant effects of cannabinoids [1,4,5,7]; conversely, animal studies have shown that a reduction in the number of CB_1_ receptors may be associated with heightened indices of anxiety and depression, especially if the disorder persists [4].
(2) Stimulation of the limbic and paralimbic areas might decrease amygdala and hypothalamus activity, regulating the hypothalamic–pituitary axis and cortisol response, and, therefore, decreasing hypervigilance and hyperarousal [1,5]. Conversely, a low eCB tone contributes to amygdala hyperactivation as well as anxiety and hyperarousal symptoms characteristic of PTSD, including sleep disturbances, memory and cognitive impairments, depression, and suicidality [14]. Interestingly, a difference in gender has been evidenced, with males showing a higher degree of endocannabinoids released in response to a stressor and stronger physiological effects to cannabis compared to women [4,26,27];
(3) Within the eCB system, reduced peripheral levels of anandamide, abnormal CB_1_ receptor-mediated anandamide signaling and compensatory increase of CB_1_ receptor availability are implicated in PTSD etiology and degree of intrusive symptoms [1,4,7,11,27,28];
(4) Cannabinoid modulation exerts effects on memory processes through alteration of the brain-derived neurotrophic factor (BDNF) concentrations in the hippocampus and the basolateral amygdala, as well as altering long-term potentiation in hippocampal neurons [27].

### 1.5. Therapeutic use of Synthetic Cannabinoids in PTSD

Several cannabinoid-acting compounds already approved to treat low appetite, nausea, vomiting, pain, and spasticity in cancer, AIDS, and multiple-sclerosis, have been furtherly developed due to the increasing interest in the role of the eCB system in fear, anxiety and stress, and in specific psychiatric conditions, such as PTSD [11]. Thus, a growing interest around the therapeutic use of cannabis and cannabinoids for the treatment of the PTSD symptoms [1] has emerged in recent years, with animal studies showing CBD may facilitate the disruption of fear memory consolidation, decrease the salience of ordinarily significant stimuli, or facilitate the extinction of fear memories [1], processes that are relevant to PTSD’s psychopathology [2,29,30]. The beneficial effects on anxiety depend on the cannabinoids’ agonism on the CB_1_ receptors, showing in animal models a biphasic dose-dependent effect, producing anxiolytic-like effects at low doses and producing an anxiogenic response at higher doses, possibly related to differences in sensitivities of CB_1_ receptors in neuronal systems [31]. Among all, Nabilone appeared to be the most explored synthetic cannabinoid in the treatment of PTSD. It is a synthetic cannabinoid agonist already approved by the Food and Drug Administration (FDA) for the treatment of chemotherapy-induced nausea and vomiting [32], but it has been also shown as promising for the treatment of PTSD-related insomnia and nightmares with increased sleep time and reduction of daytime flashbacks. Interestingly, it has also been proposed for harm reduction in cannabis dependence [28], with little evidence of development of tolerance and abuse recorded [33].

### 1.6. Aims of the Paper

Current evidence regarding the therapeutic use of CBD and synthetic cannabinoids (SC) for PTSD in humans is minimal, with mixed results, ranging from improved symptoms to cautions concerning their efficacy. To date, no clinical trials evaluating their effectiveness in reducing symptoms of PTSD in humans have been completed.

Therefore, we conducted a retrospective file review of adult patients with PTSD who were treated with CBD, nabilone, marijuana, or medical cannabis. This review firstly aims at providing an overview of PTSD and briefly describing the current knowledge available relating to cannabis, cannabinoids, and their use in PTSD treatment. Subsequently, a systematic approach including all the potential evidence examining cannabis and synthetic cannabinoids effectiveness in PTSD symptom reduction has been carried out. A review of all objectively selected, critically assessed, and reasonably synthesized evidence on experimental data, available up to May 2019, was undertaken.

## 2. Methods

### 2.1. Search Sources and Strategies

A systematic literature review was conducted following methods recommended by the Cochrane Collaboration [34], the process and results were documented in accordance with the Preferred Reporting Items for Systematic Reviews and Meta-Analyses (PRISMA) guidelines [35]. Literature searches were performed by using the following electronic databases (last update: May 2019): MEDLINE, PubMed, Cochrane Library, and Web of Science online databases. We combined the search strategy of free text terms and exploded MESH headings for the topics of cannabis, synthetic cannabinoids, and PTSD as follows: (((cannabis[Title/Abstract]) OR (synthetic cannabinoids [Title/Abstract])) AND ((PTSD[Title/Abstract]) OR (Posttraumatic stress disorder[Title/Abstract]))). The strategy was first developed in MEDLINE and then adapted for use in the other databases. No restrictions by language were included in the search strategy. Moreover, no limitations on the year of publication were applied. Thus, studies published up to 31 May 2019 were included. Further studies were retrieved through hand-searches of reference listings of relevant articles and consultation with experts in the field.

### 2.2. Study Selection

We considered studies that included cannabis and/or synthetic cannabinoid treatment for PTSD. We examined all titles and abstracts and obtained full texts of potentially relevant papers. Working independently and in parallel, two reviewers (LO and SC) read the papers and determined whether they met inclusion criteria. Duplicate publications were excluded. All English-language articles identified by the data sources, reporting original data related to cannabis and/or cannabinoids in PTSD were evaluated in the present review. All experimental and observational study designs were included apart from case reports. Randomized, controlled clinical trials involving humans were prioritized whilst preclinical/animal studies were excluded in the present systematic review. Narrative and systematic reviews, letters to the editor and book chapters were excluded as well for the specific aim of the systematic review but considered useful for the background of the research. Furthermore, only studies recruiting adult subjects were considered.

### 2.3. Data Extraction and Management

LO and SC independently extracted the data on participant characteristics, intervention details and outcomes measures. Disagreements were resolved by discussion and consensus with a third member of the team (GDP). Data were collected using a data extraction spreadsheet developed specifically for this study.

### 2.4. Characteristics of Included Studies

**Figure 1:**
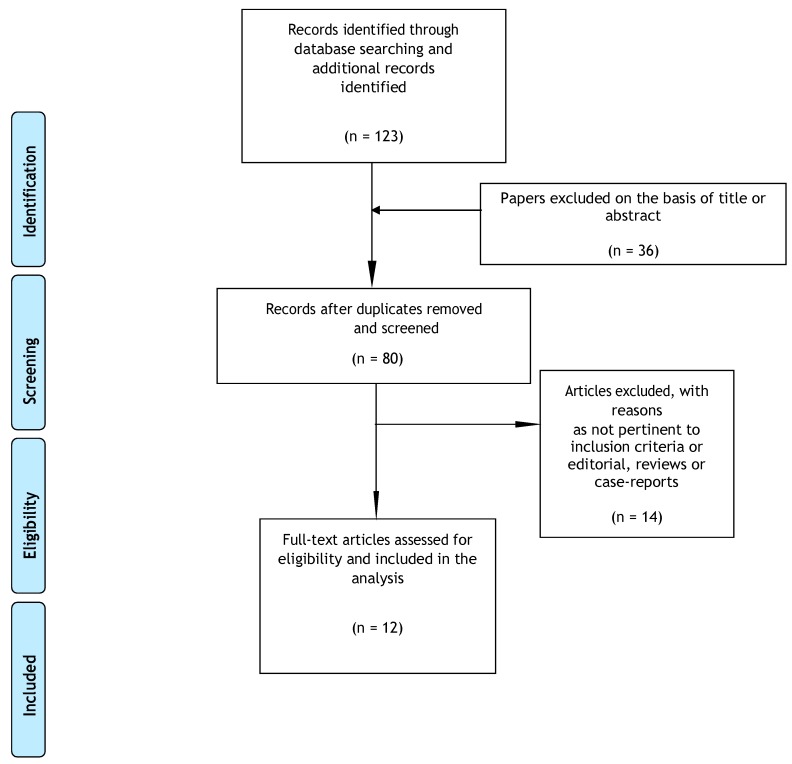
Selection of studies retrieved for the systematic review [35].

From 123 potentially relevant records from the search of databases and additional sources, 36 were excluded on the basis of title or abstract (Figure 1). The remaining 87 studies were retrieved for more detailed evaluation, of which 7 were excluded because of duplication, 66 were excluded as not pertinent to inclusion criteria or editorial, reviews or case-reports, whilst 2 were excluded due to being without full text. Overall, 12 studies met the inclusion criteria, of these: 3 about nabilone, 3 about THC, 1 about CBD, and 5 about medical cannabis were thoroughly analyzed. The main characteristics of the 12 studies are reported in Table 1.

## 3. Results

### 3.1. Medical Cannabis

A cross-sectional study of 170 medical cannabis users in California, estimated around 19% of participants with a PTSD diagnosis. Cannabis users with high PTSD scores were more likely to use cannabis to improve sleep and as coping strategy, even though only a minority (8/40) reported a significant reduction in PTSD symptomatology [14]. Conversely, another cross-sectional study from the same group recruited 217 medical cannabis users in California, reporting a reduction of hyperarousal symptoms, such as stress (24%) and anxiety (20%); depressive symptoms (10%); and in general of PTSD symptomatology (4%) amongst PTSD participants, particularly those with greater levels of traumatic intrusions and lower levels of well-being [36]. This result was echoed by a study that retrospectively evaluated PTSD symptoms described by patients following the New Mexico Medical Cannabis Program from 2009 to 2011, reporting a reduction of > 75% in PTSD symptomatology in patients using cannabis compared to when they were not [37]. Conversely, a longitudinal, observational study evaluating the association between cannabis use and PTSD symptom severity recruited 2,276 PTSD participants admitted to specialized veterans’ treatment programs, classified into four groups according to their cannabis use: those without cannabis use at admission or after discharge (“never used”); those who used cannabis at admission but not after discharge (“stoppers”); those who used at admission and after discharge (“continuing users”) and those using cannabis after discharge but not at admission (“starters”) [38], and interestingly, cannabis use was significantly associated with worse outcomes in PTSD symptom severity, violent behavior, and measures of alcohol and drug use. At follow-up, stoppers and never users had the lowest levels of PTSD symptomatology whilst starters showed the highest levels of violent behavior [38]. Finally, analyzing 202 patients coming from a SUD inpatients treatment facility with and without PTSD diagnosis [39], current PTSD was associated with greater subjective emotional reactivity to the trauma narrative only amongst subjects without cannabis dependence; amongst those with cannabis dependence, subjective emotional reactivity did not differ as a function of PTSD status. Cannabis-dependent participants (with and without PTSD) reported less subjective emotional reactivity than participants with PTSD but without cannabis dependence. No significant differences were found in cortisol reactivity [39].

### 3.2. THC

A large sample of combat-exposed U.S. veterans who used marijuana at least once per week reported significant expectations of cannabis-induced relief from PTSD symptomatology, particularly for relief from intrusive symptoms (e.g., repeated, disturbing thoughts or dreams) followed by hyperarousal (e.g., trouble sleeping and irritability), avoidance (e.g., thoughts or activities related to trauma), and numbing (e.g., feeling distant, emotionally numb) [17]. These data appeared to be consistent with the ‘self-medication’ hypothesis of cannabis use for PTSD symptoms. An open-label pilot study carried out on 10 outpatients with chronic PTSD, on stable medication, found that add-on 5 mg of THC twice a day may lead to an improvement in sleep quality and a reduced frequency in PTSD-related nightmares, even though it may cause some mild adverse effects (e.g., headache, dizziness, and dry mouth) [40]. A case-control, cross-sectional study, evaluating the association between cannabis use and PTSD symptomatology, reported no significant differences between cases and controls in mean PTSD CheckList (PCL) scores and no association between PTSD scores and frequency of cannabis use. The findings did not support the theory that cannabis would be associated with less severe PTSD symptomatology [41].

### 3.3. CBD

A retrospective open-label study examining CBD oral administration in flexible doses on PTSD symptomatology for 8 weeks reported a significant reduction in PTSD severity and intensity. CBD was well tolerated, and no patients discontinued treatment due to side-effects [25].

### 3.4. Nabilone

Fraser [33] reported a study examining the effect of nabilone in managing PTSD-related nightmares. Nabilone treatment significantly reduced the presence and intensity of nightmares as well as increased hours of sleep per night of PTSD subjects. Discontinuation was attempted every 6 months. Whilst four patients had a complete recovery after 4–12 months, the rest experienced recurrence of nightmares following nabilone withdrawal, thus necessitating resumption of treatment. A retrospective study recruiting 104 male inmates with serious mental illness showed that nabilone prescription significantly reduces polypharmacy risk and significantly improves PTSD-associated insomnia, nightmares, and PTSD symptoms [28]. A double-blind, placebo-controlled cross-over trial carried out on 29 Canadian male military service members with documented PTSD received double-blind treatment with 0.5 mg of nabilone or placebo and were followed until 7 weeks and then, following a 2-week wash-out period, were titrated with the other study treatment and followed for an additional 7 weeks, in order to evaluate the potential use of nabilone for the treatment of PTSD-related nightmares [42]. The clinician-administered PTSD scale recurring and distressing dream scores were significantly reduced in the nabilone group compared to placebo [42].

## 4. Discussion

A significant overlap has been demonstrated between PTSD and SUD [13] in several epidemiological studies which estimate that individuals with PTSD are 2–4 times more likely to have a SUD compared to non-PTSD subjects [43,44]. PTSD subjects with concomitant SUD reported being more likely to take substances to “self-medicate”, meant to mitigate distressing PTSD symptoms [45,46,47]. Particularly, expectations for anxiety and reduced tension represented the most commonly reported motivations for using cannabis and cannabinoid compounds amongst PTSD subjects [48], lifetime cannabis use being 3.3 times more likely in PTSD diagnosis [48]. Given the interactions between cannabinoids and specific neurotransmitters (e.g., GABA, serotonin, glutamate, and dopamine), it has been proposed that cannabis may potentially confer some degree of medical benefit to PTSD. In fact, PTSD subjects may use cannabis and cannabis-related compounds mainly due to their anxiolytic, sedative, hypnotic, dream recall suppressor, and antipsychotic activities [1,14,17,25,28,32,36,37,39]. Interestingly, military veterans are increasingly using cannabinoids for relief of PTSD-induced nightmares [14,28,33,40,42]. Nonetheless, nightmares associated with PTSD are often a residual symptom that remains difficult to treat despite improvements in other domains [9].

Preclinical research has shown promising findings for CBD as an enhancer of fear extinction and therapeutic consolidation of emotional memories [27,30,49]. Current evidence regarding the use of CBD for PTSD in humans and its relevance for intrusive memories and memory consolidation in PTSD is minimal and controversial [25]. A case-report reported that oral CBD administration (12–37 mg daily) was associated with reduced anxiety and sleep symptoms related to PTSD in a 10-year-old sexually-abused patient [50]. CBD has been shown to cause a decreased response to and increased extinction of aversive memories, improve performance on inhibitory avoidance tasks, as well as produce an anxiolytic effect in PTSD patients [25,51,52,53]. Preclinical and clinical studies on nabilone show promising findings, mainly in reducing nightmares and sleep disorders related to PTSD [27,28,33,42,53,54]. Conversely, no clinical evidence exists so far for dronabinol.

Despite improvements in PTSD symptomatology, there are demonstrable adverse health risks associated with cannabis use, as chronic recreational use is associated with dependence and THC-related cognition dysfunction and risk of psychosis [31,55]. Similarly, chronic use of marijuana is associated with global cortical downregulation of CB_1_ receptor availability, as well as across regions such as the temporal lobe, nucleus accumbens, and cingulate cortex, resulting in reduced endogenous cannabinoid functioning, reflecting tolerance and dependence effects [27]. Moreover, the anxiolytic effects of cannabis depend upon the neurobiological interplay or ratio metric relationship between the two major phyto-cannabinoids found in the cannabis plant, THC and CBD [11,56]. Apart from beneficial effects on anxiety, hyperarousal, and trauma-related symptoms described, synthetic cannabinoids have been reported to induce anxiety in healthy subjects and increase symptom severity in PTSD patients [38,41], effects which have been attributed to a downregulation of the eCB signaling system, resulting in tolerance to the drug’s anti-anxiety effects and, in some cases, an unmasking of pro-anxiety properties [3]. Moreover, some toxic effects, including respiratory depression, hyperthermia, acute cerebral ischemia, and seizures have been related to synthetic cannabinoids [32]. Finally, abrupt discontinuation of daily synthetic cannabinoids use can precipitate withdrawal syndrome three or four days after stoppage characterized by waxing and waning behavioral, mood, and physical symptoms such weakness, sweating, restlessness, dysphoria, sleeping problems, anxiety, craving, and diaphoresis [32].

Furthermore, several limitations should be considered when interpreting the findings coming from the present systematic review. First, studies examined here show an extreme heterogeneity of methodological strategies (in terms of sample features, sample size, pretreatment cannabis users or not, concomitant other substances abused, type of cannabinoid taken, variable and/or unspecified dosages and route of administration of cannabinoids used by PTSD patients, etc.). Second, there are, so far, no randomized, controlled, clinical trials with active marijuana use. Furthermore, for ethical reasons it is not possible to carry out a clinical trial using medical cannabis in all countries, hence, most findings presented here come only from countries in which medical cannabis is allowed and/or performing trials using THC and/or other synthetic cannabinoids is permitted.

Increasing our understanding of appropriate cannabinoids dosing levels must be further established before definitive clinical trials can begin, and this may increasingly reflect a move away from whole plant products due to difficulty in regulating levels of the active compounds in this form of the product. Furthermore, the eCS acts in different ways even in the same disease states, meaning that for many conditions the direction in which the system should be modulated is unclear and upstream options for treatment should also be researched [27]. These limitations notwithstanding, our findings highlight the importance of carrying out further research, more specifically focusing on the role of the eCS in other systems relevant to PTSD, such as the dopaminergic and serotonergic pathways.

## 5. Conclusions

Given the recent move towards understanding shared mechanisms between psychiatric fields, such as between PTSD and psychotic illnesses, there is an increasing need to deepen knowledge of the role of specific molecular mediators between these illnesses which may explain their similarities and overlapping routes, including in the field of SUD. Nowadays, the eCS represents a system where shared mechanisms may be identified. Hence, exploring its variable molecular interactions in anxiety and PTSD may benefit and improve knowledge as well in other psychiatric fields. Finally, the efficacy and safety/tolerability of both natural and recently emergent synthetic cannabinoids in the treatment of other psychiatric disorders should be better investigated, including PTSD. Randomized clinical trials and experimental studies should be furtherly encouraged due to the limited clinical evidence so far published.

## Figures and Tables

**Table 1:** Summary of retrieved papers.

Study (Author(s), Year of Publication)	Sample Characteristics	Cannabinoid or Cannabis-Based Medicine Implicated	Control Group (If Any)	Dose (s)	ROA	Outcomes Measures Used	Main Findings
Bonn-Miller et al. (2014a) [14]	170 patients at a medical cannabis dispensary in California	CBD + THC	Pts without PTSD	n.d.	n.d.	PCL-C CMMQ MSHQ AUDIT IDAS	Improvement of PTSD-related sleep disturbances but only 8/40 of PTSD subjects reported a reduction in PTSD symptoms.
Earleywine and Bolles (2014) [17]	650 combat-exposed male veterans	THC	None	unknown	Smoked, at least once per week	PCL-M CES	Significant expectations of cannabis-induced relief from PTSD symptomatology, particularly for intrusion, hyperarousal, then avoidance, and numbing.
Elms et al. (2019) [25]	11 PTSD outpatients	CBD	None	Flexible for 8 weeks	OS capsule or liquid spray once or twice per day based on severity of symptoms	PCL-5	Decreased PTSD symptom severity at 8 weeks from baseline.
Cameron et al. (2014) [28]	104 male inmates with serious mental illness	Nabilone	None	Mean initial dose: 1.4 mg daily (0.5-2 mg) Mean final dose: 5 mg daily (0.5-6 mg)	n.d.	PCL-C GAF	Significant improvement in PTSD-associated insomnia, nightmares, PTSD symptoms.
Fraser (2009) [33]	47 patients with treatment-resistant PTSD	Nabilone	None	0.5 mg titrated up to a max of 4 mg daily	OS 1 h prior to bedtime	Nightmare presence and intensity; hours of sleep	Reduction in nightmare intensity, subjective improvement in sleep time, quality of sleep, and reduction of daytime flashbacks and night sweats.
Bonn-Miller et al. (2014b) [36]	217 patients at a medical cannabis dispensary in California	CBD + THC	Pts without PTSD	n.d.	n.d.	n.d.	Reduction of stress, anxiety, depression, and PTSD symptomatology.
Greer et al., (2014) [37]	80 PTSD patients	CBD+THC	None	Unknown proportion	Smoked	CAPS	PTSD symptoms reduction.
Wilkinson et al. (2015) [38]	2276 PTSD veterans admitted to intensive PTSD treatment programs and divided into a) never cannabis users; b) cannabis users stoppers; c) continuing cannabis users; d) cannabis use starters	CBD+THC	Comparing groups	n.d.	n.d.	ASI MISS for PTSD	Starting cannabis use worsened PTSD symptoms. Stopping cannabis use improved PTSD symptoms. At follow-up, stoppers and never cannabis users had lower levels of PTSD symptoms, and starters had the highest levels of violent behavior.
Tull et al. (2016) [39]	202 subjects with and without PTSD with a co-occurring stimulant, cocaine or alcohol use disorder admitted to a SUD treatment facility	CBD + THC	Placebo	n.d.	n.d.	SCID-I CAPS MINI PANAS DUQ	Current PTSD was associated with greater subjective emotional reactivity to the trauma script only in subjects without cannabis use. Cannabis users (with and without PTSD) reported less subjective emotional reactivity than participants with PTSD but without cannabis use.
Roitman et al. (2014) [40]	10 PTSD outpatients from Israel	THC	None	2x2.5 mg daily titrated to 5 mg x 2 daily	OS 1 h after waking up and 2 h prior to bed	PSQI NFQ NES	Reduction in nightmares and improvement in sleep quality.
Johnson et al. (2016) [41]	700 veterans enrolled in the primary care mental health integration program	THC	Placebo	n.d.	Smoked	TLFB ASSIST PCL-CPHQ-9 PQSIA BOMC	No association between cannabis use and less severe PTSD symptomatology.
Jetly et al. (2015) [42]	10 male military personnel with PTSD	Nabilone	Placebo	0.5 mg titrated to 3 mg	OS 1 h prior to bed	CAPS sleep items PTSD dream rating scale Sleep diary CGI-C WBQ	Reduction in nightmares.

Abbreviations: ROA: route of administration; pts: patients; PTSD: post-traumatic stress disorder; wks: weeks; CBD: cannabidiol; OS: oral; PCL-C: PTSD Checklist-Civilian Version; PCL-5: patient-completed PTSD Checklist for the DSM-5; CMMQ: Comprehensive Marijuana Motives Questionnaire; MSHQ: Marijuana Smoking History Questionnaire; AUDIT: Alcohol Use Disorders Identification Test; IDAS: Inventory of Depression and Anxiety Scale; GAF: Global Assessment of Functioning; PCL-M: Post-Traumatic Stress Disorder Checklist-Military Version; CES: Combat Exposure Scale; PSQI: Pittsburgh Sleep Quality Index; NFQ: Nightmare Frequency Questionnaire-Revised; NES: Nightmare effects survey; CAPS: Clinical Administered PTSD Scale; CGI-C: Clinical Global Impression of Change; WBQ: General Well Being Questionnaire; n.d.: not documented; TLFB: Alcohol Timeline Followback; ASSIST: Alcohol, Smoking, and Substance Involvement Screening Test; PHQ-9: Patient Health Questionnaire; PQSIA: Paykel questionnaire for suicidal ideations and attempts; BOMC: Blessed Orientation-Memory-Concentration Test; SCID-I: Structured Clinical Interview for DSM-IV Axis; ASI: Addiction Severity Index; MISS: Mississippi Scale for PTSD; I Disorders; MINI: Mini International Neuropsychiatric Interview, version 6.0; PANAS: Positive and Negative Affect Scales; DUQ: Drug Use Questionnaire.
